# Metformin Improves Overall Survival of Colorectal Cancer Patients with Diabetes: A Meta-Analysis

**DOI:** 10.1155/2017/5063239

**Published:** 2017-02-08

**Authors:** Fanqiang Meng, Li Song, Wenyue Wang

**Affiliations:** Department of Gastrointestinal Surgery, China-Japan Friendship Hospital, Beijing 100029, China

## Abstract

*Introduction*. Diabetic population has a higher risk of colorectal cancer (CRC) incidence and mortality than nondiabetics. The role of metformin in CRC prognosis is still controversial. The meta-analysis aims to investigate whether metformin improves the survival of diabetic CRC patients.* Methods*. PubMed, EMBASE, and Cochrane Library were searched till July 1, 2016. Cohort studies were included. All articles were evaluated by Newcastle-Ottawa Scale. Hazard Ratios (HRs) with 95% confidence intervals (CIs) for each study were calculated and pooled HRs with corresponding 95% CIs were generated using the random-effects model. Heterogeneity and publication bias were assessed.* Results*. We included seven cohort studies with a medium heterogeneity (*I*^2^ = 56.1% and *p* = 0.033) in our meta-analysis. An improved overall survival (OS) for metformin users over nonusers among colorectal cancers with diabetes was noted (HR 0.75; 95% CI 0.65 to 0.87). However, metformin reveals no benefits for cancer-specific survival (HR 0.79, 95%, CI 0.58 to 1.08).* Conclusions*. Metformin prolongs the OS of diabetic CRC patients, but it does not affect the CRC-specific survival. Metformin may be a good choice in treating CRC patients with diabetes mellitus in clinical settings.

## 1. Introduction

Colorectal cancer (CRC) is the third most commonly diagnosed cancer in the world and the fourth leading cause of cancer mortality [1]. CRC survival depends on the stage at diagnosis. Localized stage CRC has a 90% 5-year survival rate, while survival decreases to 70% for regional stage and 13% for patients diagnosed at regional and distant stage [2]. Factors that benefit the prognosis of CRC patients include early diagnosis and timely and proper treatment, while heavy alcohol consumption [3], obesity [4, 5], and diabetes mellitus [6–8] are recognized as negative prognostic factors. Diabetic population has a higher risk of CRC incidence and mortality than nondiabetics [9]. A meta-analysis of 26 studies shows that all-cause mortality risk and CRC-specific mortality in CRC patients with diabetes have increased by 17% and 12%, respectively [10].

Metformin, one of the biguanide class, is a widely used drug for the treatment of diabetes. It maintains blood glucose level by decreasing liver glucose production and increasing the sensitivity to insulin of the body [11]. Given that diabetes is associated with higher cancer mortality, researchers have investigated if antidiabetic medications such as metformin would modify the negative effect. Preclinical studies found that metformin reduces insulin and IGF-1 level in serum and stables blood glucose level through AMPK pathway and thus reverses the tumor promoting effect driven by hyperinsulinemia and hyperglycemia [10, 12–15]. In clinical settings, some studies showed a protective role in terms of decreasing the CRC-specific and overall mortality, as well as prolonging the overall survival (OS) [16–18]. However, some studies did not find any associations between metformin use and the OS of CRC patients [19–21].

The relationship between metformin use and the prognosis of CRC patients with diabetes is still controversial according to the current results. In order to clarify whether metformin improves the survival of diabetic CRC patients, we conducted the meta-analysis.

## 2. Methods

### 2.1. Search Strategy and Selection Criteria

This meta-analysis is reported according to the Preferred Reporting Items for Systemic Reviews and Meta-Analyses (PRISMA) Statement.

We selected relevant studies published between Jan 1, 1980, and July 1, 2016, by searching PubMed, Cochrane, and EMBASE. No language restrictions were applied. We used MeSH terms combined with related text words and keywords. The following search string was used: ((((((((((((((Neoplasms, Colorectal) OR Colorectal Neoplasm) OR Neoplasm, Colorectal) OR Colorectal Tumors) OR Colorectal Tumor) OR Tumor, Colorectal) OR Tumors, Colorectal) OR Colorectal Carcinoma) OR Carcinoma, Colorectal) OR Carcinomas, Colorectal) OR Colorectal Carcinomas) OR Colorectal Cancer) OR Cancer, Colorectal) OR Cancers, Colorectal) OR Colorectal Cancers AND ((((((Dimethylbiguanidine) OR Dimethylguanylguanidine) OR Glucophage) OR Metformin Hydrochloride) OR Hydrochloride, Metformin) OR Metformin HCl) OR HCl, Metformin. We considered all potentially eligible studies for review.

### 2.2. Study Selection and Data Extraction

We regarded studies as eligible for inclusion if they met the following criteria: (1) cohort or retrospective cohort studies; (2) reported time to event data (Hazard Ratio (HRs) with 95% confidence interval (CI)) or number of patients in each subgroup and 5-year survival rate or any data that could be used to estimate HRs; (3) diabetes and metformin use being identified prior to colorectal cancer diagnosis. Exclusion criteria were as follows: (1) case control studies; (2) less than 6 months of metformin use; (3) median follow-up time less than 3 years.

Two independent investigators (Li Song and Fanqiang Meng) reviewed study titles and abstracts independently. Studies that met the inclusion criteria were retrieved for full-text assessment. Disagreement was resolved by discussion or by a third reviewer (Wenyue Wang). We extracted the following data from each selected study: (1) author names, year of publication, and country; (2) mean age, tumor stage, or gender composition; (3) number of patients with or without metformin use; (4) OS or cancer-specific mortality and adjusted HRs with their 95% CIs.

### 2.3. Quality Assessment

Newcastle-Ottawa statement, a “star system” scale, was used to evaluate the quality of the eligible studies mainly on three aspects: selection, comparability, and exposure [24]. All questions were assigned a score of one point, with the exception of comparability of study groups, in which a maximum of two points was awarded. Studies with a cumulative score ≥ 7 were considered to be of high quality.

### 2.4. Statistical Analyses

Pooled HRs with 95% CI were analyzed using a random-effects model if the heterogeneity was considerable, and a fixed-effects model was performed otherwise. Heterogeneity analysis was performed by calculating *I*^2^ index. We assessed the possibility of publication bias by constructing a standard funnel plot. We assessed funnel plot asymmetry using Egger tests and defined significant publication bias as a *p* value < 0.1. All statistical analyses were carried out using Stata14 software.

## 3. Results

### 3.1. Literature Search

**Figure 1:**
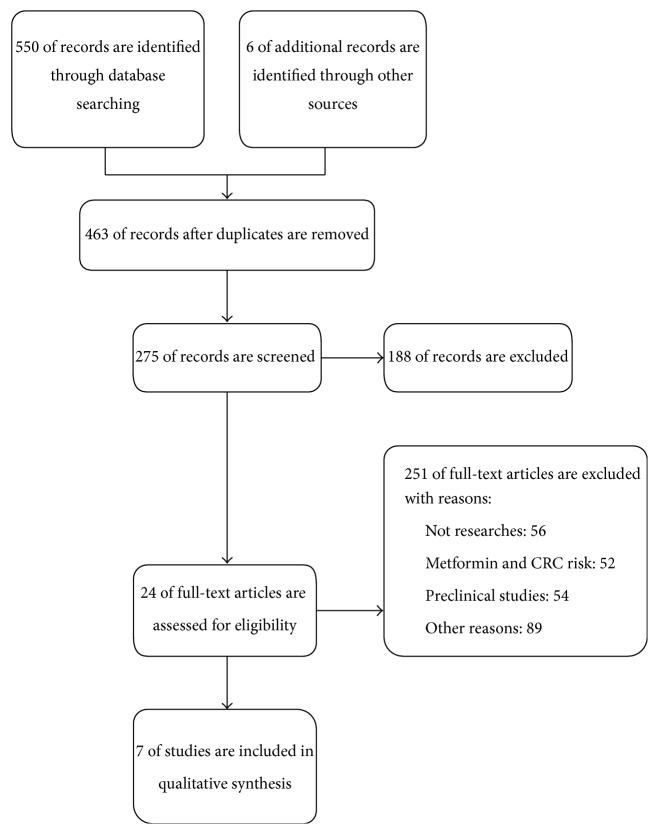
Flow diagram of studies included in the meta-analysis.

This systemic review was conducted according to the guidelines of PRISMA [25].  Figure 1 shows the searching process. A total of 556 citations were identified using PubMed/Medline and EMBASE. 463 remained after duplicates were removed. After scanning titles and abstracts, 24 articles were included for full-text reading. Finally 7 studies matched our inclusion criteria and were included in our meta-analysis (see Figure 1).

### 3.2. Study Characteristics

The characteristics of the 7 studies included are shown in Table 1 (see Table 1). These studies provided data on OS and cancer-specific mortality among colorectal cancer patients with diabetes mellitus who take metformin or other methods to control blood sugar level. The 7 studies were all cohort studies, most of which were published within 5 years. 4 studies were performed in Europe (Ireland, Denmark, Netherlands, and Northern Ireland), 2 in North America, and one study in Asia. Hazard ratio (HR) was used in most studies as the main factor. All 7 studies reported overall survival (OS) and 4 reported cancer-specific mortality (CSM). Multivariate Cox proportional hazard model, logistic regression model, or Kaplan-Meier were employed to analyze the effect of metformin use on CRC patients. Different confounding variables were adjusted in each study (mainly age, tumor stage, tumor grade, year of diagnosis, comorbidity, aspirin use, exposure to nonmetformin ADDs, socioeconomic status, radiation therapy, sex, type of tumor, chemotherapy, ASA score, blood transfusion, smoking, alcohol consumption, BMI, race, etc.).

The quality of the 7 included studies was appraised with reference to the Newcastle-Ottawa statement (see Table 2). Overall quality score ranges from 6 to 9 among the 7 studies which indicates the quality of the included study was moderate or high.

### 3.3. Pooled Effect of Metformin on Survival Outcomes of Colorectal Cancer with Diabetes

**Figure 2:**
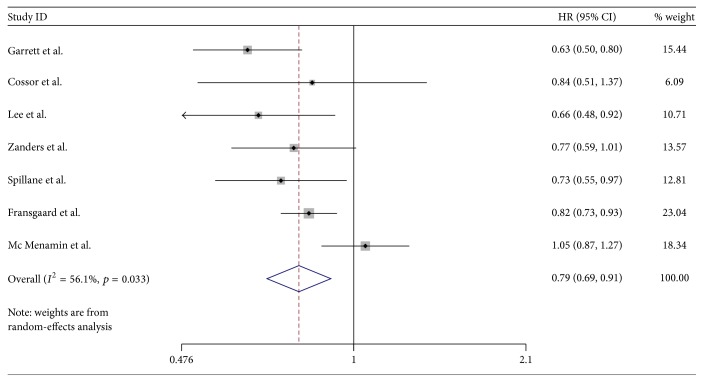
Forest plot of the association between metformin use and colorectal cancer OS.

**Figure 3:**
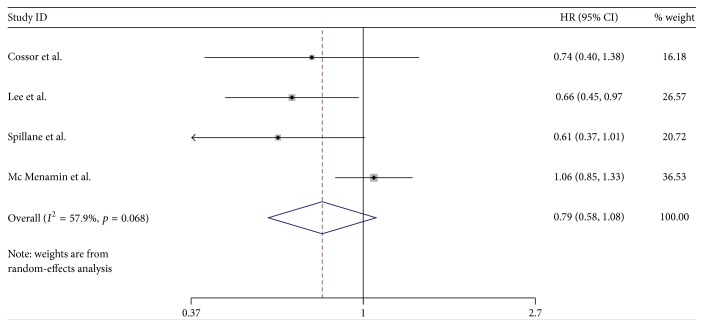
Forest plot of the association between metformin use and cancer-specific mortality.

The estimated HRs for association between exposure to metformin and colorectal cancer survival were shown in Figure 2 (see Figure 2). The Forest plot in Figure 2 portrays a series of HRs and their confidence intervals (CI) at 95%. The HR value ranged from 0.48 to 1.27. 4 studies showed an apparent protective association, while the other 3 were not statistically significant. Zanders et al. [20] and Mc Menamin et al. [21] were the two studies with the largest sample size. These two studies did not show an improved survival benefit, while the pooled results (HR 0.79; 95% CI 0.69 to 0.91) revealed an improved OS for metformin users over nonusers among colorectal cancers with diabetes. Of the 7 studies included, 4 reported cancer-specific mortality. HR value for cancer-specific mortality ranges from 0.37 to 1.38 with the pooled results (HR 0.79; 95% CI 0.58 to 1.08) (see Figure 3). Our study shows that metformin use in colorectal cancer patients with diabetes improved OS, reduced cancer-specific mortality, but was not statistically significant.

### 3.4. Publication Bias

**Figure 4:**
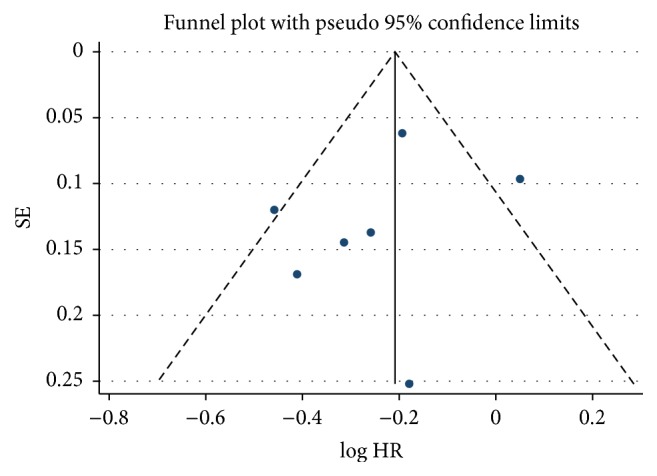
Funnel plot analysis to detect publication bias.

**Figure 5:**
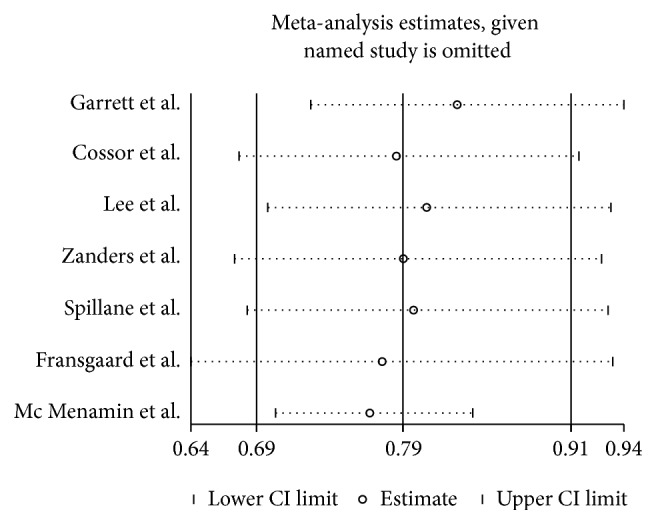
Sensitivity analysis by excluding one study each time and the pooling estimate for the rest of the studies.

In order to check heterogeneity statistically, the *Q* test and *I*^2^ statistics were applied. The results of *Q* test and *I*^2^ statistics were shown in Figure 2. We did not detect publication bias in the funnel plot (see Figure 4). Also Egger's test (*p* = 0.452) did not show publication bias statistically. Trim and fill method was applied to further estimate the effect of publication bias (see Figure 5). Sensitivity analysis shows that the removal of any individual study did not have a substantial impact on the pooled-effect estimates.

## 4. Discussion

Our meta-analysis evaluates whether metformin would affect the prognosis of diabetic colorectal cancer patients. Seven cohort studies were included in our analysis. We found that the metformin group has a prolonged OS over nonusers with a pooled HR of 0.75 (95% CI 0.65 to 0.87). Four out of seven studies conducted a pooled analysis of cancer-specific mortality. However, metformin reveals no benefits for colorectal cancer patients with diabetes mellitus in further analysis (HR 0.79, 95% CI 0.58 to 1.08).

The association among diabetes, metformin, and colorectal cancer has been widely explored. In Luo et al.'s [9] and Guraya's [26] meta-analyses, the increased CRC risk is due to hyperglycemia, hyperinsulinemia, and high IGF-1 levels, which are common in diabetic patients [13–15]. Similarly, diabetic CRC patients tend to have a worse prognosis [10]. Many studies have found that metformin, a basic antidiabetic medication, could reduce CRC incidence [27, 28]. Metformin decreases insulin and IGF-1 levels and stables blood sugar level by activating AMP-activated protein kinase (AMPK), thus preventing tumor cells from growing [10, 12, 29]. Activated AMPK could phosphorylate tumor suppressor tuberous sclerosis complex 2 (TSC2) to inhibit mTOR activity, which plays a critical role in cancer progression [29]. The inhibition of mTOR provides a rationale for metformin to suppress tumor growth. Moreover, metformin antitumor mechanisms include its cytotoxic effect on cancer stem cells and synergist effect with chemotherapeutic drugs [30, 31].

Our meta-analysis shows that CRC patients with diabetes will benefit from metformin in terms of OS but not CRC-specific survival (CS). In a previous meta-analysis of six cohort studies by Mei et al., metformin group had a better OS (HR 0.56, 95% CI 0.41–0.77) and a better CS (HR 0.66, 95% CI 0.50–0.87) than nonusers [32]. The favoring HR of OS was confirmed in another pooled analysis by He et al., but they did not analyze the relationship between metformin and CRC-specific mortality [28]. Compared with the two previous studies, we exclude meeting abstracts that cannot be evaluated by Newcastle-Ottawa statement Quality Assessment Criteria and include some high-quality cohort studies published in 2015-2016, which may explain the result difference among the three meta-analyses. In our analysis, the result that metformin decreases only all-cause mortality but not CRC-specific mortality could be explained by metformin decreasing diabetes-specific and cardiovascular-specific mortality in CRC patients [33].

Our analysis has a medium heterogeneity of *I*^2^ = 56.1% and *p* = 0.033. To further analyze the heterogeneity, we performed funnel plot and Egger's test to evaluate publication bias. Sensitivity analysis is also conducted, showing that removing any of the seven studies would not affect the association between metformin and OS of diabetic CRC patients.

Our study has several limitations. First, we did not analyze whether nonmetformin antidiabetic medications influence the survival of diabetic CRC patients. Given that diabetic patients may take more than one medication to control blood glucose level or use metformin and insulin or insulin analogs at the same time, there may exist some cofounding factors. Compared with metformin users, more nonmetformin users may take insulin to control glucose level. Diabetic patients using insulin are considered to be more severe than those who take oral medications. Therefore, insulin use may be an important confounding factor which affects the overall survival. The studies included did not provide enough data about insulin use among patients, so we do not know whether insulin use could change the overall survival. Second, studies that we included in the meta-analysis did not give details about how long or how many pills a day the participants take metformin. Therefore, we could not evaluate whether a dose-dependent benefit exists. Third, tumor stage is an important factor concerning overall survival. Spillane et al. [17] included patients from stage I to stage III, while all other studies included stage I~IV patients. Most researches do not provide more specific data about how much metformin affects diabetic patients with early, advanced, or metastatic stages, respectively, so the analysis of metformin effect in different tumor stages is impossible. Fourth, the two studies with most participants did not show the protective effect of metformin among CRC patients, even though sensitivity analysis was conducted. Zanders et al. [20] and Mc Menamin et al. [21] studies were all published recently, so we still need to pay more attention to the emerging evidence of this topic. Moreover, some confounding variables such as physical activity, BMI, and exposure to nonmetformin ADDs were not well adjusted for included studies. Finally, all the studies included were observational studies, which have methodical shortcomings and are prone to time-related biases. This may overestimate the effect of metformin among diabetic CRC patients.

In conclusion, our meta-analysis shows that metformin, the commonly used antidiabetic medication, prolongs the overall survival of diabetic CRC patients, but it does not affect the CRC-specific survival in those people. Metformin may be a better choice than other antidiabetic medications when treating CRC patients with diabetes mellitus. However, further studies, especially well-designed cohort studies or RCTs, are expected.

## Figures and Tables

**Table 1:** Baseline characteristics of included studies in the meta-analysis.

Author	Year	Country	Stage	Range	Follow-up period	Age	Metformin users	Metformin nonusers	Outcome assessment	Confounding variables adjusted
Spillane et al. [17]	2013	Ireland	I–III	2001–2006	1194 person-year	75	207	108	OS, CS	1,2,3,4,5,6,7,8,9
Zanders et al. [20]	2015	Netherlands	I–IV	1998–2011	3.4 years	73.2	666	377	OS	1,2,3,4,6,7,9,10,11,12,
Cossor et al. [19]	2013	USA	I–IV	2005–2010	4.1 years	71	84	128	OS, CS	1,2,
Fransgaard et al. [22]	2015	Denmark	I–IV	2003–2012	5 years	72.3	1962	388	OS	1,2,5,10,11,13,14,15,16,17
Menamin et al. [21]	2015	Northern Ireland	I–IV	1998–2009	4 years	72.1	675	552	OS, CS	2,4,5,6,7,9,10,11,12
Garrett et al. [23]	2012	USA	I–IV	2004–2008	NA	62.7	208	216	OS	1,2,4,17,18
Lee et al. [16]	2011	Korea	I–IV	2000–2008	3.5 years	62.9	258	337	OS, CS	2,4,5,6,7,10,15,19

1: age, 2: tumor stage, 3: tumor grade, 4: year of diagnosis, 5: comorbidity, 6: aspirin use, 7: exposure to nonmetformin ADDs, 8: socioeconomic status, 9: radiation therapy, 10: sex, 11: type of tumor, 12: chemotherapy, 13: ASA score, 14: blood transfusion, 15: smoking, 16: alcohol consumption, 17: BMI, 18: race, and19: HbA1c.

**Table 2:** Methodological quality of cohort studies included in the meta-analysis.

	Quality assessment criteria								Overall quality score (Max = 9)
	selection				Comparability	Outcome			
	Representativeness of the exposed cohort	Selection of the nonexposed cohort	Ascertainment of exposure	Demonstration that outcome of interest was not present at start of study	Comparability of cohorts on the basis of the design or analysis	Assessment of outcome	Was follow-up long enough for outcomes to occur	Adequacy of follow-up of cohorts	
Spillane et al.	*✴*	*✴*	*✴*	*✴*	*✴✴*	*✴*	*✴*	*✴*	9
Zanders et al.	*✴*	*✴*	*✴*	*✴*	*✴✴*	*✴*	*✴*	*✴*	9
Cossor et al.	*✴*	*✴*	*✴*	*✴*	*✴*	*✴*	—	—	6
Fransgaard et al.	*✴*	*✴*	*✴*	*✴*	*✴✴*	*✴*	*✴*	*✴*	9
Menamin et al.	*✴*	*✴*	*✴*	*✴*	*✴✴*	*✴*	*✴*	*✴*	9
Garrett et al.	*✴*	*✴*	*✴*	*✴*	*✴*	*✴*	*✴*	*✴*	8
Lee et al.	*✴*	*✴*	*✴*	*✴*	*✴✴*	*✴*	—	*✴*	8
